# Integrating Constituents Absorbed into Blood, Network Pharmacology, and Quantitative Analysis to Reveal the Active Components in *Rubus chingii* var. *suavissimus* that Regulate Lipid Metabolism Disorder

**DOI:** 10.3389/fphar.2021.630198

**Published:** 2021-06-29

**Authors:** Man-jing Jiang, Wan-fang Huang, Shuai Huang, Yi-xiang Lu, Yong Huang, Pei-lin Du, Yao-hua Li, Lan-lan Fan

**Affiliations:** ^1^ School of Pharmacy, Guangxi University of Chinese Medicine, Nanning, China; ^2^ Department of Pharmacy, Wuhan University of Bioengineering, Wuhan, China; ^3^ Guangxi Institute for Food and Drug Control, Nanning, China

**Keywords:** *Rubus chingii* var. *suavissimus*, UPLC-Q/TOF-MS, chemical profile, constituents absorbed into blood, network pharmacology, lipid metabolism disorders, quantification

## Abstract

*Rubus chingii* var. *suavissimus* (S. K. Lee) L. T. Lu (RS)—a sweet plant also known as Tiancha distributed in the south of China where it is used as a beverage—recently gained extensive attention as adjuvant therapy of diabetes and hypertension. Although pharmacological studies indicate that RS has beneficial effects in regulating lipid metabolism disorder characteristics, the active chemicals responsible for this effect remains unclear. The present study aims to predict the effective substances of RS on regulating lipid metabolism disorder through the analysis of the chemical profile of RS, the absorbed prototype components in rat plasma, and network pharmacology. Also, a UPLC method able to quantify the screened potential effective chemicals of RS products was established. First, a total of 69 components—including diterpene, triterpenoids, flavonoids, polyphenols, and lignans—were systematically characterized in RS. Of those, 50 compounds were detected in the plasma of rats administered with RS extract. Through network pharmacology, 9 potential effective components, 71 target genes, and 20 pathways were predicted to be involved in RS-mediated regulation of lipid metabolism disorder. The quantitative analysis suggested that the contents of potential effective components varied among samples from different marketplaces. In conclusion, the presented results provide a chemical basis for further research of *Rubus chingii* var. *suavissimus*.

## Introduction

Lipid metabolism disorder is an important pathogenic factor of diseases characterized by an abnormal lipid metabolism, such as atherosclerotic vascular disease, type 2 diabetes, and nonalcoholic fatty liver disease (NAFLD) (Guo et al., 2017; Zhang et al., 2018). While statins are widely used as classical chemical medicines in the treatment of lipid metabolism disorder, they have side effects such as rhabdomyolysis and digestive problems (Jones and Davidson, 2005; Thompson et al., 2016). Recent observations indicating that some ethnomedicines and natural products have lipid-lowering effects with multitarget, multilink, and slight side effect features resulted in the development of combination therapies with western medicines for the treatment of lipid metabolism disorder (Zhang et al., 2018; Li et al., 2020).

*Rubus chingii* var. *suavissimus* (S. K. Lee) L. T. Lu (Flora of China, 2003) or *Rubus suavissimus* S. Lee (RS) (Li, 1981) also known as Tiancha or sweet tea is a sweet plant distributed in the south of China where it is used as a beverage and the adjuvant therapy of diabetes, the treatment of hypertension, and urinary tract infections (Guangxi Food And Drug Administration, 2011). As an ideal sugar substitute and low-calorie, low-toxic healthcare product, it is comparable with *Siraitia grosvenorii* (Swingle) C. Jeffrey ex Lu et Z. Y. Zhang and *Stevia rebaudiana* Bertoni (Liang et al., 2003) and is mainly used as a tea drink or sweetener with medicinal characteristics. For example, RS was shown to improve blood lipid regulation due to its richness of a variety of active ingredients (Ezure and Amano, 2011; Koh et al., 2011; Wang et al., 2015). Rubusoside—the sweetness compound of RS accounting for up to 5%—was shown to be able to lower serum total cholesterol (TC), triglycerides (TG) levels, and lipid peroxides (Sun et al., 2001; Tian et al., 2001). Our previous study showed that both RS (Jiang et al., 2021) and rubusoside (Li et al., 2020) could alleviate high-fat-diet–induced lipid metabolism disorder and liver injury in a golden hamster model. Besides, RS-derived polyphenols could reduce abdominal fat, triglycerides (TG), and total cholesterol (TC) in animal models of high-fat diet (Liu et al., 2014; Wang et al., 2015). Moreover, RS-derived flavonoids could regulate lipid metabolism disorder by scavenging free radicals and preventing lipid peroxidation (Liu et al., 2017). However, the systematic chemical basis of RS and the identification of the active compounds active in mediating the lipid metabolism disorder should be further evaluated.

Analysis of compounds migrating into the blood proved to be a very useful approach for the identification of bioavailable components of traditional Chinese medicine (TCM) (Zhang et al., 2019). Moreover, the combined analysis of compound characterization and blood absorbent compounds is generally accepted for mining the potential biological active constituents of TCM (Bi et al., 2018). With the development of bioinformatics, network pharmacology has become a novel approach to systematically predict and reveal the active components, molecular targets, and action mechanisms of TCM from the molecular level to the pathway level by establishing a multilevel network model of “components−targets−diseases” (Chen et al., 2016).

Therefore, this study aims to predict the active components and molecular mechanisms underlying the protective action of RS on regulating lipid metabolism disorder. First, an ultra-perfomance liquid chromatography with quadrupole time-of-flight tandem mass spectrometry (UPLC-Q/TOF-MS) method was established to characterize the chemical profile of RS as well as the *in vivo* absorbed components from RS extract-administrated rats. Based on this, a network pharmacology approach was developed to systematically predict the main active components and possible molecular action mechanisms. In addition, the main chemicals predicted to have the potential activity to regulate lipid metabolism disorder were quantified by a validated UPLC method. These results provide a reference for further research and exploration of pharmacodynamics material basis and mechanisms of RS.

## Materials and Methods

### Materials and Reagents

Thirteen batches of plant materials from markets were collected (Table 3) and authenticated as the leaves of *Rubus chingii* var. *suavissimus* (S. K. Lee) L. T. Lu by Professor Yi Cai, Guangxi University of Chinese Medicine. The voucher specimen is deposited in the herbarium of School of Pharmacy, Guangxi University of Chinese Medicine.

Gallic acid, caffeic acid, ellagic acid, rutin, hyperoside, isoquercitin, quercitrin, quercetin, kaempferol, rubusoside, steviol, isosteviol, ursolic acid, and oleanolic acid (purity ≥ 98%) were provided by Chengdu PFID Co., Ltd. Formic acid and acetonitrile of LC-MS grade were purchased from Thermo Fisher Scientific. Other chemicals were of analytical grade.

### Animals

Male Sprague Dawley (SD) rats (200 ± 20 g) were obtained from Hunan SJA Laboratory Animal Co., Ltd. (Hunan, China). Animal care and procedures were approved by and conducted according to the standards of the Guangxi University of Chinese Medicine (Nanning, China) (Certificate DW20200713-003). The rats were housed in an animal room (22 ± 2°C, 60 ± 10% relative humidity) on a 12 h dark/12 h light cycle with food and water *ad libitum*. Before experiments, they were fasted for 24 h but with free access to water.

### Reference Stock Solutions

Accurately weighted references of caffeic acid, rutin, hyperoside, isoquercitin, quercitrin, quercetin, kaempferol, rubusoside, steviol, isosteviol, ursolic acid, and oleanolic acid were dissolved in methanol, except for gallic acid (in water) and ellagic acid (in DMSO) to get the stock solutions of each reference compound. All reference stock solutions were stored at 4°C.

### Chemical Profile of RS by UPLC-Q/TOF-MS

#### Preparation of Sample Solutions

0.5 g weighed sample powders (RS08) were extracted with 10 mL 60% ethanol at room temperature for 60 min by ultrasonication, then complemented for the weight loss, and centrifuged at 12,000 rpm for 10 min, after which the supernatant fluid was diluted 10 times with 60% ethanol and filtered through a 0.22 μm filter before analysis.

#### Preparation of Reference Working Solutions

Each reference stock solution was mixed and diluted with methanol to a suitable concentration and then filtered through a 0.22 μm filter before analysis.

### Animal Experiment and Plasma Sample Preparation

#### Preparation of RS Extract for Intragastric Administration to Rats

200.0 g RS powder (RS08) was extracted three times with 1,600 mL 95% ethanol (60 min per time) by ultrasonication, filtered, and recycled the solvent under reduced pressure to obtain the dried extract. It was diluted by 0.5% sodium carboxymethyl cellulose (CMC-Na) to prepare the suspension for animal experiments.

#### Preparation of Plasma Sample

Ten male SD rats were randomly divided into control group (two) and RS group (eight). Rats in the RS group were gave at a fourfold human equivalent dosage (4 × 2.06 g/kg), and the blood was collected from the orbital veins in heparinized centrifuge tube at 0.17, 0.33, 0.5, 1, 1.5, 2, 4, 6, 8, and 12 h. Rats in the control group received an equal volume of 0.5% CMC-Na orally after which the blood was collected by the abdominal aortic method. The blood samples were centrifuged at 4,000 r/min for 10 min at 4°C to obtain the plasma which was stored at −80°C.

50 μL plasma samples of the above time points were combined (or 500 μL control plasma samples) and loaded onto a pre-equilibrated solid-phase extraction (SPE) column and successively eluted by 3 ml of water and 3 ml of methanol. The methanol eluent was collected and evaporated to dryness under nitrogen at 40°C. The residue was reconstituted in 100 μL methanol and centrifuged at 12,000 rpm for 10 min to obtain the supernatant fluid for analysis.

#### UPLC-Q/TOF-MS Analysis Conditions

The UPLC-Q/TOF-MS analysis was performed on a Dionex UltiMate3000 coupled with a hybrid quadrupole orthogonal time-of-flight (Q-TOF) tandem mass spectrometer (Bruker Daltonik GmbH, Bremen, Germany). The chromatographic separation was carried out on an InertSustain ODS-3 LC column (2 µm, 2.1 × 150mm, GL Sciences Inc.). The mobile phase consisted of  0.1% formic solution (A) and acetonitrile (B) using the following gradient program: 0–1 min, 5% B; 1–6 min, 5–15% B; 6–20 min, 15–24% B; 20–25 min, 24–33% B; 25–30 min, 33–54% B; 30–35 min, 54–80% B; 35–38 min, 80% B; 38–40 min, 80–95% B; 40–43 min, 95% B; 43–45 min, 95–5% B; and 45–50 min, 5% B with a sample injection volume of 5 μL. The solvent flow rate was 0.5 ml/min and the column temperature was set at 40°C.

The ESI source was operated at an optimized capillary voltage of 3.5 V (ESI^–^), with a drying gas flow and temperature of 8 L/min at 200°C, a nebulizer pressure of 2.0 bar (=29 psi), a collision RF of 500 Vpp, a transfer time of 60.0 µs, and a prepulse storage of 5 µs. Automatic MS/MS experiments were performed adjusting the collision energy values as follows: *m/z* 50, 10 eV; *m/z* 500, 25 eV; and *m/z* 1,000, 40 eV. Argon was used as collision gas for CID in the MS^E^ mode. The ion scan ranged from *m/z* 50 to 1,000. Sodium formate solution was used as a calibration solution. The MS data were processed through DataAnalysis 4.3 software (Bruker Daltonics, Bremen, Germany).

### Network Pharmacology-Based Analysis

#### Bioactive Components of RS and Potential Targets

According to the results of the chemical profiling and components absorbed in plasma of RS, the absorbed prototypes in rat plasma were selected as candidate compounds. The molecular targets of RS were identified by PharmMapper (http://www.lilab-ecust.cn/pharmmapper/), STITCH (http://stitch.embl.de/) and SwissTargetPrediction (http://www.swisstargetprediction.ch/). Beyond this, some known molecular targets of identified compounds were collected for comprehensive analysis through literature mining. All targets were combined and the duplicates were removed.

#### Screening of Putative Therapeutic Targets

The related targets of lipid metabolism disorder were collected from GeneCards (https://www.genecards.org/). Next, the overlapping genes between RS and lipid metabolism disorder were matched by the Venn diagram.

#### Network Construction of Interactions Between RS and Lipid Metabolism Disorder

To gain insight into the molecular mechanisms underlying the action of RS on lipid metabolism disorder, a bioactive compound–therapeutic target network was constructed with Cytoscape 3.7.1 to depict the interactions between target genes and bioactive compounds. Next, the degree value was calculated by using the NetworkAnalyzer plug-in. Compounds with a high degree value were deliberated as main bioactive RS-derived compounds potentially involved in the RS-mediated alleviation of lipid metabolism disorder. The protein–protein interaction (PPI) network was supported by STRING (https://string-db.org/). For the analysis, the study organism was limited to *Homo sapiens*, while all other settings were kept as default values.

#### Enrichment of KEGG Pathways

To decipher the possible signaling pathway involved in the intervention of RS on lipid metabolism disorder, the KEGG pathway enrichment analysis was performed with DAVID 6.8 (https://david.ncifcrf.gov/); terms with *p* < 0.05 were considered significant and captured.

### Quantitative Analysis of RS by UPLC

#### Preparation of Sample Solutions

0.5 g weighed sample powders were extracted with 10 ml 60% ethanol at room temperature for 60 min by ultrasonication, then complemented for the weight loss, and filtered through a 0.22 μm filter before analysis.

#### Preparation of Reference Working Solution

Gallic acid, caffeic acid, rutin, ellagic acid, rubusoside, and kaempferol stock solutions were diluted into suitable working solutions with corresponding solvents before analysis.

#### UPLC Conditions

UPLC analysis was performed on an Agilent 1290 I system equipped with a 1290 VWD detector, a 1,290 vial sampler, and a 1290 high-speed pump. The column configuration was an InertSustain C_18_ LC column (2 µm, 3.0 × 150 mm, GL Sciences Inc., Japan). The mobile phase consisted of 0.1% phosphoric acid (A) and acetonitrile (B) using a gradient program of 0–6 min, 5–15% B; 6–20 min, 15–24% B; 20–37 min, 24–80% B; 37–38 min, 80–95% B; 38–38.5 min, 95–5% B; and 38.5–40 min, 5% B. The solvent flow rate was 0.35 ml/min and the column temperature was set at 40°C. The detection wavelengths were the following: 205 nm for 0–6 min; 254 nm for 6–23 min; and 205 nm for 23–40 min. An aliquot of 1.0 μL was injected for analysis.

#### Method Validation

Stock reference solutions of gallic acid, caffeic acid, rutin, ellagic acid, rubusoside, and kaempferol were prepared and diluted to six appropriate concentrations using the corresponding solvent to get the reference working solutions. Six calibration curves were constructed by plotting the content (Y) vs. the peak areas (X) of each analyte. The limit of quantitation (LOD) and limit of detection (LOQ) were determined at a signal-to-noise ratio (S/N) of about 3 and 10, respectively. The intraday precision was examined by six repetitive injections within one day, and the interday precision was carried out for three consecutive days. Repeatability was acquired by analyzing six replicates of the same sample solution (RS08). To determine the stability, the same sample solution (RS08) was injected at 0, 1, 2, 4, 6, 8, 12, and 24 h, respectively. The accuracy of the method was expressed by the recovery. Approximately 100% amount of each reference standard equivalent to those in 0.25 g extracts were added to the same RS sample (RS08), after which the sample solutions were prepared in six replicates. Recoveries were calculated by the following equation: recovery (%) = 100 × (detected amount−original amount)/spiked amount.

### Statistical Analysis

All data were expressed as the mean ± standard deviation (SD). T-test was used to compare the two groups. The multigroup comparisons were analyzed by one-way analysis of variance (ANOVA) with SPSS 24.0 (SPSS Inc., Chicago, IL, United States).

## Results and Discussion

### Characterization of Chemical Profile of RS by UPLC-Q/TOF-MS

**FIGURE 1:**
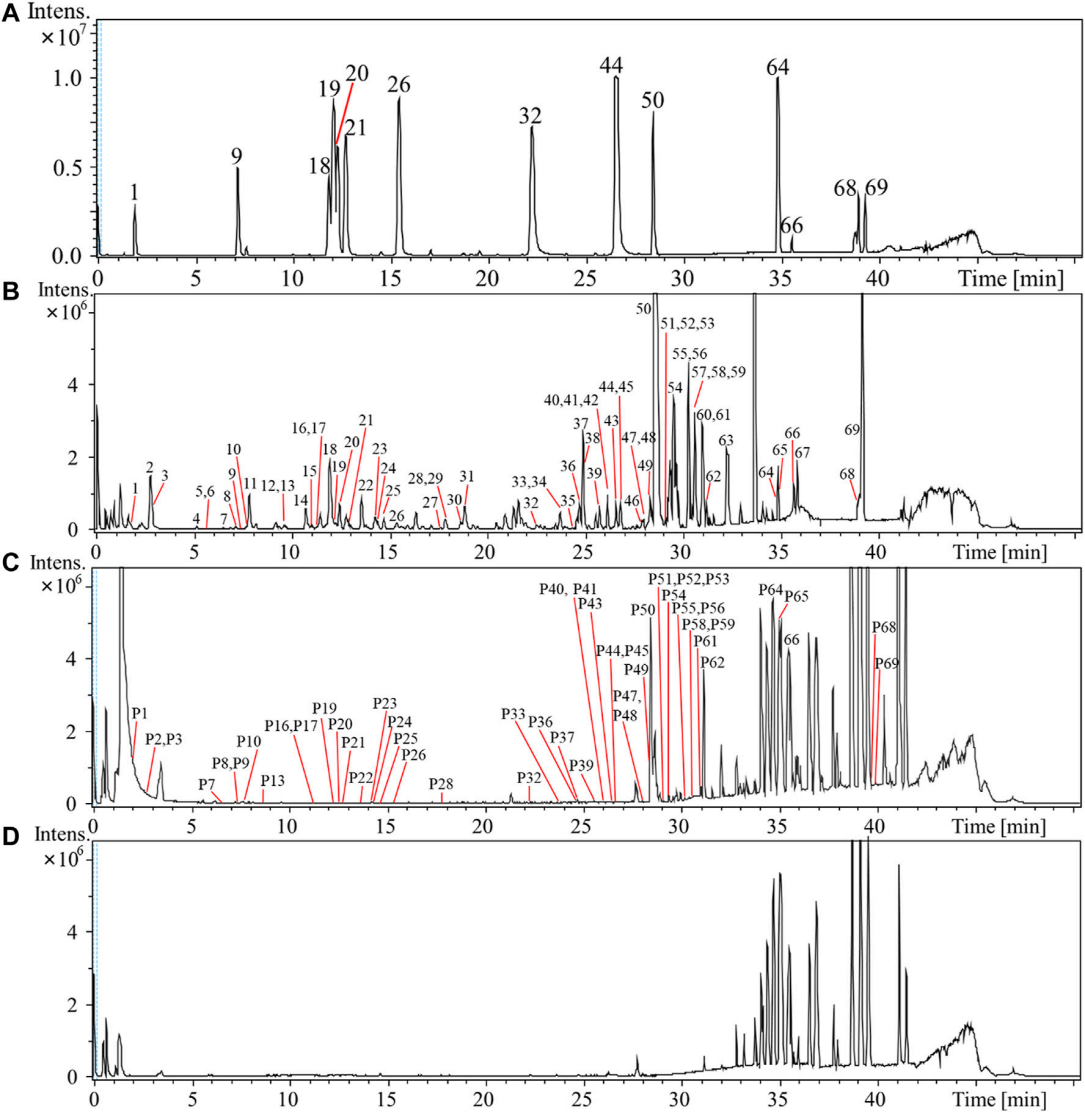
Base peak intensity (BPI) chromatogram of mixed reference compounds **(A)** and 60% ethanol extract sample of *Rubus chingii* var. *suavissimus* (RS08) **(B)**, rat plasma sample **(C)**, and blank rat plasma **(D)** of rats in negative ion mode by UPLC-Q/TOF-MS.

A UPLC-QTOF/MS method was established to characterize the chemical profile. As MS and fragmentations were most informative in the negative ion mode, all analyses were conducted in the negative ion mode. The base peak intensity (BPI) chromatogram of RS in the negative ion mode is shown in Figure 1. The identification data are summarized in Table 1, and the temporarily identified structures are shown in Supplementary Figure S1. The obtained retention time, quasimolecular ions, and the MS^n^ fragments data were compared with reference compounds, a self-built chemical database, and reported literature. In addition, a ClogP value was calculated by ChemDraw 11.0 (CambridgeSoft, United States) to estimate the retention time of the isomers.

**TABLE 1:** Characterization of chemical components of *Rubus chingii* var. *suavissimus* by UPLC-Q/TOF-MS.

No.	t_R_ (min)	Formula	PI pred. (Da)	PI meas. (Da)	Error (ppm)	Major fragment ions	Identification	ClogP	Chemical type
**1** ^a^	**1.9**	**C**_**7**_ **H**_**6**_ **O**_**5**_	**169.0142**	**169.0143**	**−0.3**	**125.0260**	**Gallic acid**	–	**P**
**2**	**2.7**	**C**_**8**_ **H**_**8**_ **O**_**5**_	**183.0299**	**183.0298**	**0.3**	**169.0139, 125.0244**	**Methyl gallate**	–	**P**
**3**	**2.8**	**C**_**7**_ **H**_**4**_ **O**_**6**_	**182.9935**	**182.9941**	**−3.4**	**139.0035, 95.0142**	**2-Pyrone-4,6-dicarboxylic acid**	–	**P**
4	5.2	C_27_H_22_O_18_	633.0733	633.0733	0.0	481.0601, 300.9988, 257.0076, 238.9998	Corilagin	0.41	P
5	5.7	C_34_H_24_O_22_	783.0686	783.0679	0.9	481.0596, 300.9992, 275.0200	Pedunculagin	–	P
6	5.7	C_27_H_22_O_18_	633.0733	633.0735	**−**0.3	300.9983, 257.0086, 229.0129, 169.0137	1-α-Galloyl-2,3-(*S*)-hexahydroxydiphenoyl-*D*-glucose	0.47	P
**7**	**6.5**	**C**_**15**_ **H**_**18**_ **O**_**9**_	**341.0878**	**341.0878**	**0.0**	**179.0354, 161.0435, 161.0254, 135.0466**	**Caffeic acid-*O-*hexoside**	–	**P**
**8**	**7.1**	**C**_**27**_ **H**_**30**_ **O**_**17**_	**625.1410**	**625.1415**	**−0.8**	**463.0863, 301.0342, 283.0229, 255.0294**	**Quercetin-3,4’-*O*-*D*-*β*-glucopyranoside**	–	**F**
**9** ^a^	**7.2**	**C**_**9**_ **H**_**8**_ **O**_**4**_	**179.0350**	**179.0351**	**−0.9**	**135.0456**	**Caffeic acid**	–	**P**
**10** ^b^	**7.6**	**C**_**21**_ **H**_**20**_ **O**_**9**_	**415.1035**	**415.1038**	**−0.9**	**161.0434, 151.0401, 123.0431**	**Toringin**	–	**F**
**11**	**7.8**	**C**_**13**_ **H**_**8**_ **O**_**8**_	**291.0146**	**291.0149**	**−1.0**	**247.0258, 203.0360, 175.0403, 147.0461**	**Brevifolincarboxylic acid**	–	**P**
12	8.6	C_21_H_10_O_13_	469.0049	469.0048	0.1	316.9907, 300.9985	Sanguisorbic acid dilactone	–	P
**13**	**8.6**	**C**_**16**_ **H**_**20**_ **O**_**9**_	**355.1035**	**355.1034**	**0.2**	**193.0511, 161.0457, 161.0239, 133.0296**	**Ferulic acid-*O-*hexoside**	–	**P**
14	10.7	C_26_H_28_O_16_	595.1305	595.1310	**−**0.9	301.0334, 271.0251, 151.0036	Quercetin-3-*O*-xylosyl glucoside	–	F
15^b^	10.8	C_27_H_30_O_16_	609.1461	609.1463	**−**0.3	447.0909, 285.0413	Cyanidin-3-*O*-sophoroside	**−**1.33	P
**16** ^b^	**11.2**	**C**_**27**_ **H**_**30**_ **O**_**16**_	**609.1461**	**609.1466**	**−1.6**	**447.0907, 285.0401**	**Cyanidin-3,5-diglucoside**	**−1.21**	**P**
**17** ^b^	**11.2**	**C**_**16**_ **H**_**14**_ **O**_**4**_	**269.0819**	**269.0816**	**1.2**	**145.0316, 105.0537**	**5-Hydroxy-7-methoxydihydroflavone**	–	**F**
**18** ^a^	**11.9**	**C**_**14**_ **H**_**6**_ **O**_**8**_	**300.9990**	**300.9995**	**−1.5**	**282.9963, 257.0091, 239.0008**	**Ellagic acid**	–	**P**
**19** ^a^	**12.2**	**C**_**27**_ **H**_**30**_ **O**_**16**_	**609.1461**	**609.1469**	**1.4**	**301.0337, 283.0230, 271.0254, 255.0293, 151.0040**	**Rutin**	–	**F**
**20** ^a^	**12.4**	**C**_**21**_ **H**_**20**_ **O**_**12**_	**463.0882**	**463.0893**	**−2.4**	**301.0339, 283.0244, 271.0251, 255.0305**	**Hyperoside**	–	**F**
**21** ^a^	**12.7**	**C**_**21**_ **H**_**20**_ **O**_**12**_	**463.0883**	**463.0877**	**0.1**	**301.0331, 283.0340, 271.0266, 255.0291**	**Isoquercitrin**	–	**F**
**22** ^b^	**13.6**	**C**_**27**_ **H**_**30**_ **O**_**15**_	**593.1512**	**593.1514**	**−0.4**	**447.0905, 285.0399, 255.0306, 151.0041**	**Cyanidin 3-*O*-rutinoside**	**−0.83**	**P**
**23**	**14.2**	**C**_**20**_ **H**_**18**_ **O**_**11**_	**433.0776**	**433.0776**	**0.0**	**301.0337, 271.0253, 255.0300, 151.0042**	**Guaijaverin**	–	**F**
**24**	**14.3**	**C**_**21**_ **H**_**20**_ **O**_**11**_	**447.0933**	**447.0937**	**−0.9**	**285.0406, 255.0303, 151.0048**	**Kaempferol-*O*-hexoside**	**0.26**	**F**
**25**	**14.6**	**C**_**27**_ **H**_**30**_ **O**_**15**_	**593.1512**	**593.1513**	**−0.2**	**285.0407, 255.0304, 151.0056**	**Kaempferol-3-*O-*rutinoside**	**−0.76**	**F**
**26** ^a^	**15.3**	**C**_**21**_ **H**_**20**_ **O**_**11**_	**447.0933**	**447.0936**	**−0.7**	**283.0241, 271.0256, 255.0297, 151.0049**	**Quercitrin**	–	**F**
27	17.5	C_20_H_18_O_11_	433.0776	433.0784	**−**1.7	301.0350, 283.0259, 255.0293	Quercetin-3-*O-α*-*D*-ribofuranoside	–	F
**28**	**17.8**	**C**_**21**_ **H**_**20**_ **O**_**11**_	**447.0933**	**447.0931**	**0.4**	**285.0413, 255.0293, 151.0044**	**Luteoloside**	**0.81**	**F**
29	17.8	C_30_H_26_O_14_	609.1250	609.1254	**−**0.7	447.0930, 285.0413, 161.0255	Kaempferol-*O*-caffeoyl-hexoside	–	F
30	18.5	C_30_H_26_O_15_	625.1199	625.1201	**−**0.3	463.0882, 301.0348, 271.0246, 255.0312	Quercetin-3-*O*-sophoroside	**−**1.86	F
31	18.8	C_30_H_26_O_15_	625.1199	625.1299	0.0	463.0888, 301.0349, 255.0291, 161.0258	Quercetin-*O*-caffeoyl-hexoside	0.89	F
**32** ^a^	**22.3**	**C**_**15**_ **H**_**10**_ **O**_**7**_	**301.0354**	**301.0353**	**0.4**	**283.0252, 271.0252, 255.0290, 151.0042**	**Quercetin**	–	**F**
**33**	**23.6**	**C**_**26**_ **H**_**42**_ **O**_**10**_	**513.2705**	**513.2705**	**0.1**	**351.2166, 333.2069,161.0460**	**Glaucocalyxin G**	**0.74**	**D**
34	23.6	C_31_H_38_O_11_	585.2341	585.2344	**−**0.5	497.1587, 493.1896, 221.0471	Erythro-7,8-dihydro-buddlenol B	1.13	L
35	24.2	C_30_H_26_O_13_	593.1301	593.1296	0.8	447.0892, 285.0398, 255.0304, 161.0255	Kaempferol-*O*-coumaroyl-hexoside	–	F
**36**	**24.6**	**C**_**20**_ **H**_**30**_ **O**_**4**_	**333.2071**	**333.2074**	**−0.9**	**315.1978, 289.2153, 271.2063**	**7*β*-Hydroxysteviol**	**2.11**	**D**
**37**	**24.7**	**C**_**26**_ **H**_**42**_ **O**_**10**_	**513.2705**	**513.2701**	**0.9**	**351.2183, 333.2087, 271.2073, 161.0454**	**Cussovantoside A**	**0.98**	**D**
38	24.8	C_31_H_38_O_11_	585.2341	585.2341	0.1	497.1587,493.1924,221.0469	Threo-7,8-dihydro-buddlenol B	1.13	L
**39**	**25.6**	**C**_**26**_ **H**_**42**_ **O**_**8**_	**481.2807**	**481.2807**	**0.1**	**319.2278, 301.2155, 161.0461**	**Sugereoside**	**1.98**	**D**
**40**	**26.0**	**C**_**26**_ **H**_**40**_ **O**_**9**_	**495.2600**	**495.2603**	**−0.6**	**333.2063,285.1886**	**7*β*,17-Dihydroxy-ent-kaur-15-en-19-oic acid 19-*O-β-D *-glucopyranoside ester**	–	**D**
**41**	**26.0**	**C**_**26**_ **H**_**42**_ **O**_**9**_	**497.2756**	**497.2761**	**−0.9**	**335.2231, 317.2139**	**7*β*,17-Dihydroxy-16*β*-ent-kauran-19-oic acid 19*-O-β- D*-glucopyranoside ester**	**1.95**	**D**
42	26.0	C_32_H_52_O_14_	659.3284	659.3280	0.6	497.2774, 335.2231	*β-D-*Glucopyranosyl 17-hydroxy-ent-kauran-19-oate-16-*O*-*β*-*D*-glucopyranoside	–	D
**43**	**26.4**	**C**_**20**_ **H**_**32**_ **O**_**5**_	**351.2177**	**351.2182**	**−1.6**	**333.2055, 303.1952**	**ent-3*α*,16*β*,17-Trihydroxy-kauran-19-oic acid**	–	**D**
**44** ^a^	**26.6**	**C**_**15**_ **H**_**10**_ **O**_**6**_	**285.0405**	**285.0406**	**−0.6**	**285.0409, 255.0306, 151.0029**	**Kaempferol**	–	**F**
**45**	**26.6**	**C**_**26**_ **H**_**44**_ **O**_**8**_	**483.2963**	**483.2958**	**1.2**	**321.2433, 285.2233, 161.0463**	**Suavisoside-A**	–	**D**
46	27.8	C_30_H_48_O_7_	519.3327	519.3328	**−**0.2	501.3219, 459.3135, 453.3014	2*β*,3*β*,19*α*,23,24-Pentahydroxy-urs-12-en-28-oic acid	3.84	T
**47**	**28.0**	**C**_**20**_ **H**_**30**_ **O**_**4**_	**333.2071**	**333.2070**	**0.3**	**315.1976, 289.2179, 271.2068**	**ent-13,17-Dihhydroxy-kauran-15-en-19-oic acid**	**2.87**	**D**
**48**	**28.0**	**C**_**38**_ **H**_**60**_ **O**_**18**_	**803.3707**	**803.3704**	**0.3**	**641.3183, 479.2645, 317.2160**	**13-[(*O*-*β*-*D*-Glucopyranosyl)oxy**]**ent-kaur-16-en-19-oic acid-2*-O*-*β*-*D*-glucopyranosyl-*β*-*D*-glucopyranosyl ester**	–	**D**
**49**	**28.2**	**C**_**26**_ **H**_**42**_ **O**_**8**_	**481.2807**	**481.2804**	**−0.7**	**319.2235, 301.2261, 161.0448**	**17-*O-β-D*-Glucopyranosyl-16*α*-ent-kauran-19-oic acid**	**4.40**	**D**
**50** ^a^	**28.4**	**C**_**32**_ **H**_**50**_ **O**_**13**_	**641.3179**	**641.3184**	**−0.9**	**479.2674, 317.2130, 273.2242, 255.2304, 161.0452**	**Rubusoside**	–	**D**
**51**	**29.1**	**C**_**20**_ **H**_**32**_ **O**_**4**_	**335.2228**	**335.2231**	**−1.1**	**335.2232, 291.2298, 243.2121**	**16*α*,17-Dihydroxy-kauran-19-oic acid**	–	**D**
**52**	**29.1**	**C**_**30**_ **H**_**46**_ **O**_**7**_	**517.3171**	**517.3180**	**−1.8**	**499.3071, 481.2952, 473.3265, 469.2952**	**Ganoderic acid C2**	–	**T**
**53**	**29.1**	**C**_**30**_ **H**_**48**_ **O**_**7**_	**519.3327**	**519.3333**	**−1.2**	**501.3205, 471.3101, 459.3106, 441.3022**	**Platycodigenin**	**4.00**	**T**
**54**	**29.4**	**C**_**30**_ **H**_**48**_ **O**_**6**_	**503.3378**	**503.3388**	**−2.0**	**485.3283, 473.3269, 455.3131, 437.3027, 419.2949**	**Terminolic acid**	–	**T**
**55**	**30.1**	**C**_**30**_ **H**_**48**_ **O**_**6**_	**503.3378**	**503.3377**	**0.3**	**485.3278, 473.3264, 455.3182, 419.2935**	**2*α*,3*β*,19*α*,23-Tetrahydroxy-urs-12-en-28-oic acid**	**4.51**	**T**
**56**	**30.1**	**C**_**20**_ **H**_**32**_ **O**_**4**_	**335.2228**	**335.2231**	**−1.0**	**317.2020, 291.2333, 243.2108**	**ent-16*β*,17-Dihdyroxy-kauran-19-oic acid**	–	**D**
57^b^	30.4	C_36_H_56_O_11_	633.3750	633.3743	1.1	501.3222, 457.3286, 429.3257	Rubuside J	–	T
**58**	**30.4**	**C**_**26**_ **H**_**42**_ **O**_**9**_	**497.2756**	**497.2755**	**0.2**	**335.2207, 317.2120, 287.2046**	**Paniculoside Ⅳ**	**2.02**	**D**
**59**	**30.4**	**C**_**26**_ **H**_**40**_ **O**_**8**_	**479.2650**	**479.2654**	**−0.8**	**317.2120, 273.2214, 255.2104**	**ent-Kauran-16-en-19-oic-13*-O-β-D*-glucoside**	**3.23**	**T**
60	30.7	C_30_H_48_O_6_	503.3378	503.3370	1.7	485.3254, 473.3212, 455.3153	2*α*,3*β*,19*α*,23-Tetrahydroxy-olean-12-en-28-oic acid	4.51	T
**61**	**30.8**	**C**_**30**_ **H**_**46**_ **O**_**6**_	**503.3222**	**501.3225**	**−0.7**	**483.3121, 467.3145**	**Ilexgenin A**	–	**T**
**62**	**31.0**	**C**_**26**_ **H**_**40**_ **O**_**8**_	**479.2650**	**479.2640**	**2.2**	**317.2124, 273.2215, 255.2104**	**Cussoracosides E**	**3.45**	**D**
63^b^	32.0	C_30_H_48_O_5_	487.3429	487.3432	**−**0.6	441.3345, 423.3220	Euscaphic acid	–	T
**64** ^a^	**34.7**	**C**_**20**_ **H**_**30**_ **O**_**3**_	**317.2122**	**317.2116**	**2.0**	**273.2226, 255.2178**	**Steviol**	–	**D**
**65**	**34.8**	**C**_**20**_ **H**_**32**_ **O**_**3**_	**319.2279**	**319.2270**	**2.7**	**289.2195, 273.2236**	**ent-16*β*, 17-Dihydroxy-kauran-3-one**	–	**D**
**66** ^a^	**35.3**	**C**_**20**_ **H**_**30**_ **O**_**3**_	**317.2122**	**317.2122**	**0.0**	**273.2214, 255.2131**	**Isosteviol**	–	**D**
67	35.5	C_30_H_48_O_4_	471.3480	471.3488	**−**1.8	471.3476, 453.3369, 435.3142	2*α*,3*β*-Dihdyroxy-urs-12-en-oic acid	–	T
**68** ^a^	**38.7**	**C**_**30**_ **H**_**48**_ **O**_**3**_	**455.3531**	**455.3545**	**−3.1**	**411.3476, 393.3508**	**Ursolic acid**	–	**T**
**69** ^a^	**39.0**	**C**_**30**_ **H**_**48**_ **O**_**3**_	**455.3531**	**455.3534**	**−0.7**	**411.3599, 393.3541**	**Oleanolic acid**	–	**T**

Note: the component absorbed into blood circulation is highlighted in bold.

a The compounds were identified by comparing with reference substances.

b The compounds were first reported in *Rubus chingii* var. *suavissimus*. F: flavonoids; P: polyphenols; D: diterpenoids; T: triterpenoids; L: lignans.

In total, 69 compounds were identified or tentatively characterized, including 20 diterpenes, 13 triterpenes, 19 flavonoids, 15 polyphenols, and 2 lignans. Compounds 1, 9, 18, 19, 20, 21, 26, 32, 44, 50, 64, 66, 68, and 69 were identified as gallic acid, caffeic acid, ellagic acid, rutin, hyperoside, isoquercitrin, quercitrin, quercetin, kaempferol, rubusoside, steviol, isosteviol, ursolic acid, and oleanolic acid through the comparison of the retention time and quasimolecular ions with reference compounds, respectively. Compounds 4 (corilagin), 19 (rutin), and 50 (rubusoside) were taken as examples for explaining the fragmentation process (Supplementary Figure S2).

Kaurane-type diterpenoids, oleanane-type triterpenoids, ursane-type triterpenoids, flavone, dihydroflavone, anthocyanidins, and flavonol—all widely described in RS—are sometimes present in the form of glycoside with one or several sugar moieties (Hirono et al., 1990; Lv and Wang, 2007; Ohtani et al., 1992; Peng et al., 2019; Wang and Lv, 2008). Upon analysis of the MS/MS spectra of kaurane-type diterpenoids and oleanane, or ursane-type triterpenoids, it was observed that a series of diagnostic ions was caused by the loss of –H_2_O, –OCH_2_, –CO_2,_ and –C_6_H_9_O_5_ (glucose unit). The main characteristic fragmentation behaviors of RS-flavonoids were the loss of glycosyl, elimination of small neutral fragments such as H_2_O and CO, and retro Diels–Alder (RDA) cleavage. RS-derived polyphenols are mainly galloyl-oxygen-diphenyl-type ellagitannins and gallotannins of which the fragmentation patterns could mainly be summarized as successive elimination of the galloyl group and lactonization (Nakano et al., 2017; Schulza and Chim, 2019).

Noteworthy, based on the reported chemicals of *Rubus L.* species or other plants (Zuo et al., 2008; Li et al., 2009; Li et al., 2010; Zhang et al., 2018; Schulza and Chim, 2019), the current study is the first to report the presence of toringin (10), cyanidin-3-*O*-sophoroside (15), cyanidin-3,5-diglucoside (16), 5-hydroxy-7-methoxydihydroflavone (17), cyanidin 3-O-rutinoside (22), rubuside J (57), and euscaphic acid (63) in *Rubus chingii* var. *suavissimus*.

### Analysis of *in Vivo* Absorbed Components in Plasma of RS Extract-Administered Rats

#### Optimization of the Methods to Collect and Pretreat Plasma

Considering that the time to being absorbed into the blood is different for each RS extract-derived component, plasma samples were first collected at 10 different time points (0.17 ∼ 12 h) after the administration of RS extract and then finally combined. An SPE column was used for protein precipitation and separation.

#### Identification of Absorbed Components in Rat Plasma Samples after Oral Administration of RS Extract

Based on reference compounds and the chemical profile of RS and by comparing pre- and postdose rat plasma, an *in vivo* absorbed prototype component profile of the plasma of RS extract-administrated rats was obtained and analyzed.

As shown in Figure 1 and Table 1, a total of 50 compounds—including 19 diterpenoids, 8 triterpenoids, 13 flavonoids, and 10 polyphenols—were identified or tentatively identified in rat plasma samples after the oral administration of RS extract. The detailed identified information, including observed mass values of quasimolecular ions, mass error, and MS^n^ fragments data, is listed in Supplementary Table S1. For example, based on the observation that P50 exhibited a parent [M–H]^–^ ion at *m/z* 641 which yielded characteristic fragment ions at *m/z* 479 [M–H–glucose]^–^, 317 [M–H–2glucose]^–^, and 273 [M–H–2glucose–CO_2_]^–^, it could be identified as rubusoside through comparison of the MS behavior to the reference.

Most diterpenoids and triterpenoids existed as unchanged prototypes in rat plasma and displayed high peak intensity in the BPI chromatogram. Compared to flavonoid aglycones, more flavone glycosides were detected. The fact that RS-polyphenols displayed low peak intensity or were even hard to detect might be due to their bioavailability and the liver first-pass effect (D'Archivio et al., 2010; Zhang et al., 2016).

### Network Pharmacology-Mediated Prediction of Effective Component and Molecular Mechanisms Underlying the Beneficial Effect of RS on Lipid Metabolism Disorder

#### Candidate Compounds and Target Genes in RS

First, 50 candidate compounds were selected based on the *in vivo* absorbed components profile of RS. Then, a total of 1,882 molecular targets were obtained from PharmMapper, STITCH, and SwissTargetPrediction. Next, the name of the identified targets was converted into an official gene symbol by UniProt (https://www.uniprot.org/). Finally, a target library with 521 target genes was established after integrating the results and removing the duplicates.

#### Acquisition of Therapeutic Targets of Lipid Metabolism Disorder

1,112 targets (relevance score>22.01) related to lipid metabolism disorder were gathered from GeneCards. Through Venn diagram matching, 213 overlapping target genes between RS and lipid metabolism disorder-related targets were identified. Of note, a target with a higher relevance score as calculated by GeneCards indicated a higher pertinence to the disease. As such, the top 71 targets (Table 2) selected by GeneCards were defined as hub genes and retained for subsequent research. Finally, 36 compounds interacting with those previously defined hub genes were identified.

**TABLE 2:** Top 71 directly relevant targets of *Rubus chingii* var. *suavissimus* in lipid metabolism disorder based on GeneCards.

No.	Target	Score	No.	Target	Score
1	LPL	114.478	37	XDH	54.72941
2	PPAR*γ*	112.1502	38	APOA2	54.1526
3	TNF	99.24533	39	OTC	54.0331
4	COMT	96.67764	40	GSTP1	53.983
5	CYP2D6	96.11501	41	CYP19A1	52.922
6	ALB	93.87442	42	ALDH2	52.37643
7	PPAR*α*	88.45335	43	AR	52.34957
8	ACE	83.09168	44	CFTR	51.83479
9	CYP3A4	80.83278	45	TLR4	51.41657
10	SLC6A3	72.99746	46	MPO	50.38863
11	MAOA	71.15596	47	G6PD	49.77814
12	HMGCR	71.08429	48	PTGS2	49.11167
13	APP	70.99548	49	CYP1B1	49.00745
14	SERPINE1	69.47766	50	MTOR	48.67168
15	ESR1	68.98495	51	GSR	47.65023
16	SNCA	66.55721	52	FABP2	46.94385
17	TP53	66.24002	53	VEGFA	45.86672
18	INSR	66.18439	54	NR3C2	45.62914
19	DRD4	66.08241	55	CYP17A1	45.42647
20	CYP2C9	64.10754	56	HSD11B1	44.66399
21	CYP1A2	63.93973	57	NR1H3	44.37109
22	DRD2	63.63854	58	PIK3CA	44.30278
23	F2	62.81121	59	SOD2	44.21383
24	SREBP1	62.42875	60	CES1	43.65325
25	AKT1	61.84262	61	MAPK1	43.44899
26	MAPT	61.83129	62	TTR	43.18982
27	NR1H2	61.45977	63	ACC1	43.12664
28	JAK2	61.03323	64	PAH	43.05243
29	CNR1	58.8269	65	ALOX5	42.59163
30	GBA	58.24603	66	FAS	42.50793
31	NOS3	58.20886	67	FTO	42.29441
32	ABCB1	57.0409	68	SCD	42.01818
33	VDR	56.72137	69	PTPN11	41.92701
34	NR3C1	55.69765	70	SELE	41.70531
35	NR1H4	55.29206	71	aP2	41.33099
36	PPARD	54.7986	–	–	–

#### Interaction Network Construction

**FIGURE 2:**
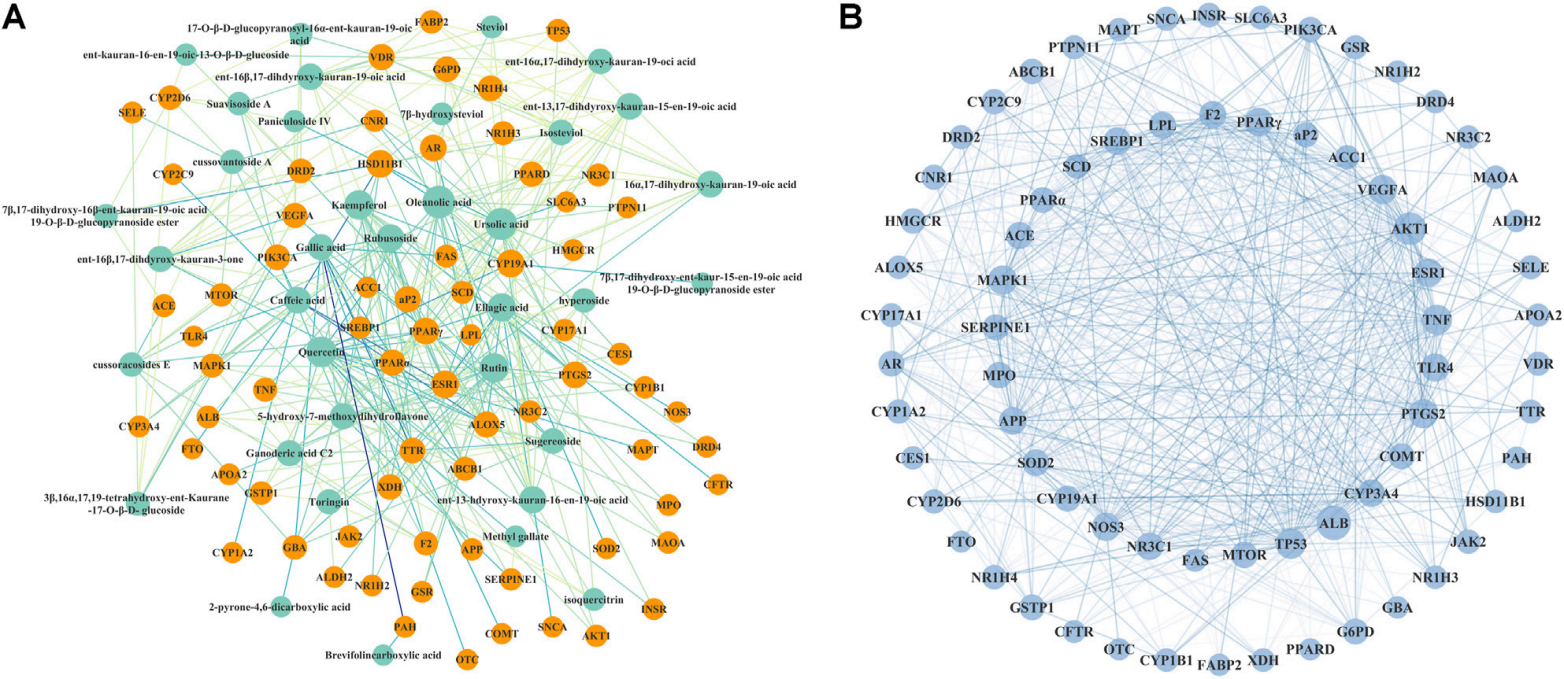
Interaction network of *Rubus chingii* var. *suavissimus* on lipid metabolism disorder. **(A)** Bioactive compound–therapeutic target network. 36 green nodes represent bioactive compounds in RS, and 71 orange nodes represent the lipid metabolism disorder-related targets of RS. **(B)** Protein–protein interaction network of the picked 71 target genes.

As shown in Figure 2A, a network linking 36 bioactive compounds and 71 target genes was visualized by Cytoscape 3.7.1. The green nodes represent bioactive compounds and the orange nodes represent putative therapeutic targets. Besides, a protein–protein interaction network (Figure 2B) illustrates the interconnection of the 71 hub targets.

This network suggested the involvement of the 36 compounds and 71 genes in the beneficial effect of RS on lipid metabolism disorder. In network analysis, a high degree value generally indicates the node is closely connected with other target genes. Based on the calculated results from NetworkAnalyzer, compounds with a degree value ≥ 11 were considered as potential bioactive ingredients. As such, nine compounds including ursolic acid (21.0), oleanolic acid (21.0), quercetin (18.0), kaempferol (16.0), gallic acid (14.0), ellagic acid (14.0), caffeic acid (11.0), rubusoside (14.0), and rutin (16.0) might be the main effective RS-derived compounds involved in the beneficial effect of RS on lipid metabolism disorder.

#### KEGG Pathway Enrichment Analysis

**FIGURE 3:**
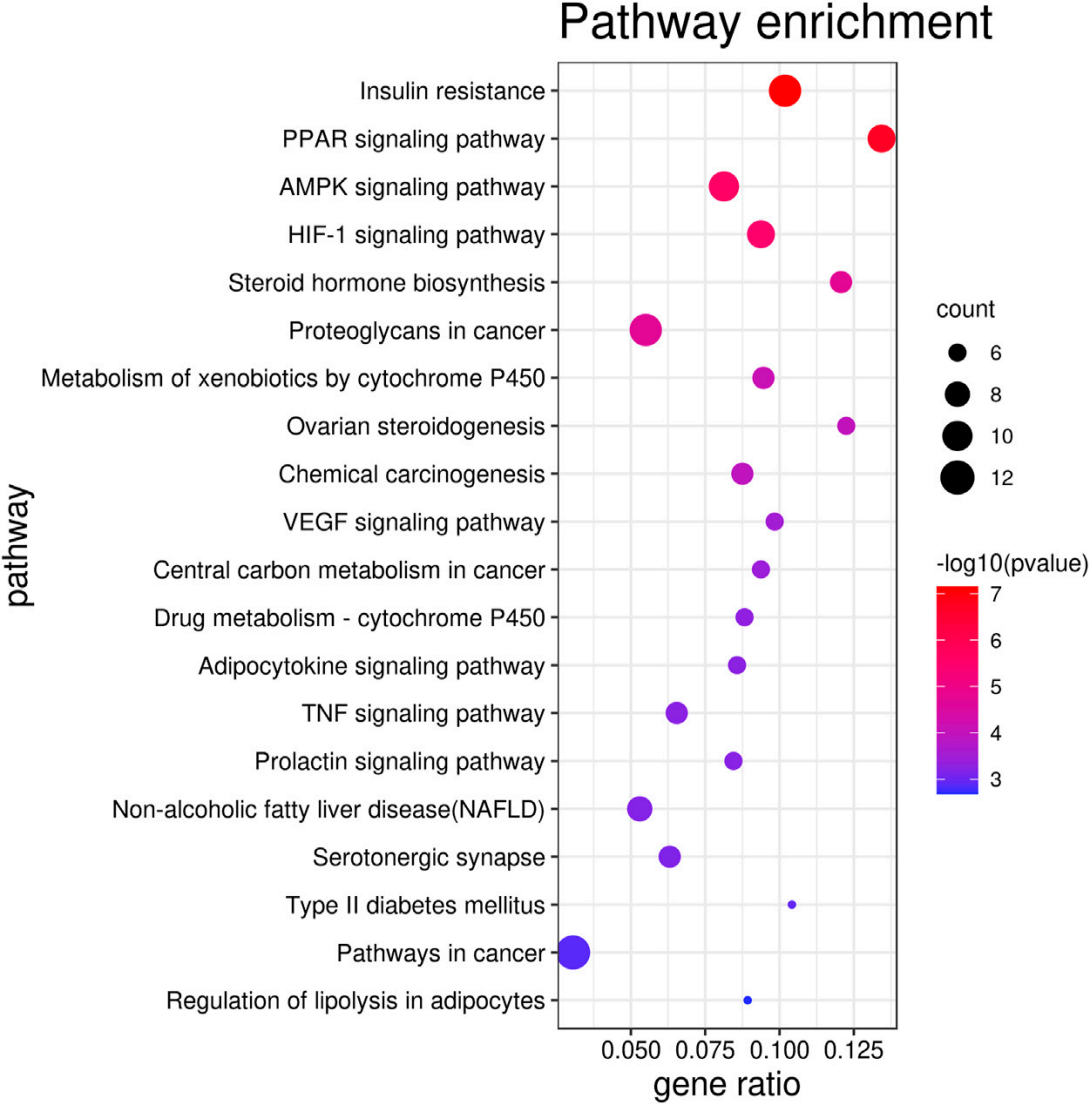
Top 20 KEGG pathways selected based on the p value.

The top 20 KEGG pathways (*p* < 0.01) are presented in Figure 3 and Supplementary Table S2 and include insulin resistance (*p* = 7.02 E^−08^, count = 11), the PPAR signaling pathway (*p* = 2.18 E^−07^, count = 9), the AMPK signaling pathway (*p* = 2.49 E^−06^, count = 10), and the HIF-1 signaling pathway (*p* = 3.55 E^−06^, count = 7).

#### Comprehensive Analysis of the Compounds, Target Genes, and Signaling Pathways Involved in the Beneficial Effect of RS on Lipid Metabolism Disorder

The network pharmacology analysis suggested that nine potential effective components (ursolic acid, oleanolic acid, quercetin, kaempferol, gallic acid, ellagic acid, caffeic acid, rutin, and rubusoside), 71 target genes, and 20 pathways are directly related to lipid metabolism disorder.

Previously, the potential of the above compounds on the regulation of lipid metabolism disorder and antiobesity have been shown. For example, ursolic acid and oleanolic acid have regulatory effects on the absorption, synthesis, and metabolism of triglycerides and cholesterol (Zhang and Shen, 2015). Also, intake of gallic acid can be beneficial for the suppression of HFD-induced dyslipidaemia, hepatosteatosis, and oxidative stress in rats (Hsu and Yen, 2007). Of note, gallic acid was previously shown to be partially responsible for the antiangiogenic (Liu et al., 2006) and antiobesity (Koh et al., 2011) activities of *Rubus* leaf. Also, caffeic acid ameliorates hepatic steatosis and reduces endoplasmic reticulum stress in obese mice by regulating autophagy (Kim et al., 2018). Rutin, quercetin, and kaempferol can alleviate high-fat diet–induced obesity and fatty liver insulin resistance (Gao et al., 2013; Bai et al., 2014; Wang et al., 2020). Our previous study indicated that rubusoside effectively alleviated liver injury of golden hamsters on a high-fat diet by reversing the metabolic pathway disorder such as amino acid metabolism and synthesis of ketone bodies (Li et al., 2020).

The interaction network indicated that RS might mediate lipid metabolism disorder through a multicomponents and multitargets approach, suggesting a synergistic effect between these compounds; however, this should be further analyzed in future studies.

The pathway enrichment analysis noted that RS might regulate multiple pathways involved in lipid metabolism disorder, such as insulin resistance and PPAR and AMPK signaling pathways. When lipid metabolism disorder occurs, excess free fatty acids (FFA) are deposited primarily in the liver and *β*-cells are injured potentially resulting in insulin resistance (IR). Meanwhile, IR could promote the transcription of fatty acid synthetase (FAS) (Meex and Watt, 2017). On the other hand, the PPAR signaling pathway is involved in adipocyte differentiation, fatty acid conversion, and lipid synthesis. PPAR*γ* is one of the key transcriptional activators of adipocyte genes such as glucose transporter 4 (Glut4), lipoprotein lipase (LPL), and fatty acid binding protein 2 (aP2). PPAR*α* and AMPK play important roles in regulating the processes of FFA transport and lipid oxidative metabolism (Madrazo and Kelly, 2008; Lopaschuk et al., 2010). Hypoxia-inducible factor-1*α* (HIF-1*α*) mainly improves abnormal lipid metabolism by regulating oxidative stress (Ma et al., 2017). Interestingly, LPL, PPAR*γ*, and PPAR*α*—genes which are directly related to the PPAR signaling pathway—are among the top 10 hub targets identified in the present study.

Our previous study showed that RS could alleviate lipid metabolism disorder in a high-fat diet golden hamster model and upregulate the expression of the PPAR pathway mediators PPAR*α* and PPAR*γ*, resulting in the activation of downstream adipocyte genes aP2, Glut4, and LPL (Jiang et al., 2021). These findings verified that RS might regulate lipid metabolism disorder through mediating the PPAR signaling pathway.

### Determination of the Content of Compounds with a Potential Effect on Lipid Metabolism Disorder in Different RS Samples by UPLC

In the current study, network pharmacology identified nine compounds—including ursolic acid, oleanolic acid, quercetin, kaempferol, gallic acid, ellagic acid, caffeic acid, rubusoside, and rutin—as potential bioactive ingredients of RS involved in mediating lipid metabolism disorder. However, ursolic acid and oleanolic acid could not be detected in the RS samples due to their weak UV absorptions whereas the content of quercetin was below the detection limit. Therefore, six compounds—gallic acid, caffeic acid, rutin, ellagic acid, rubusoside, and kaempferol—were chosen to determine the content in RS samples obtained from multiple marketplaces (RS01-RS13).

#### Optimization of Extraction and UPLC Chromatographic Conditions

First, several variables including solvent, extraction methods, extraction time, and number of cycles were optimized to obtain the maximal extraction efficiency. Maximal extraction was achieved through ultrasonication with 20 volumes of 60% ethanol for 60 min once or eight volumes of 95% ethanol for three times in case of intragastric administration to rats.

Next, the conditions for chromatographic analysis including the type of column, mobile phase gradients, and detection wavelength were optimized. The results showed that optimal separation was obtained by eluting RS samples on an InertSustain C_18_ LC column at 40°C using a linear gradient of 0.1% phosphoric acid and acetonitrile within 40 min. Considering that the maximum absorption wavelength of rubusoside situates at 205 nm while that of the other references situates at 254 nm, a wavelength conversion program was used to obtain a better resolution.

#### Method Validation of UPLC

The regression equation, linearity range, correlation coefficients, and LODs and LOQs of gallic acid, caffeic acid, rutin, ellagic acid, rubusoside, and kaempferol are summarized in Supplementary Table S3. All calibration curves indicated good linearity (*r* ^2^ ≥ 0.9993) within the test ranges while the overall LODs and LOQs were ranged from 0.09 to 1.00 ng and 0.30 to 3.00 ng, respectively. The RSDs of precision, repeatability, stability, and recovery are shown in Supplementary Table S4. For six analytes, the RSDs of intra- and interday precisions of these analytes were less than 1.50 and 1.93%, respectively. The reproducibility RSDs were less than 2.94%. The RS sample solution was stable within 24 h with an RSD of less than 1.89%. The recoveries ranged from 97.58 to 100.29%, and the recovery RSDs ranged from 1.21 to 2.24%. Based on these data, the developed UPLC method was considered reliable, accurate, and suitable for the quantification of those components.

#### Sample Analysis

**FIGURE 4:**
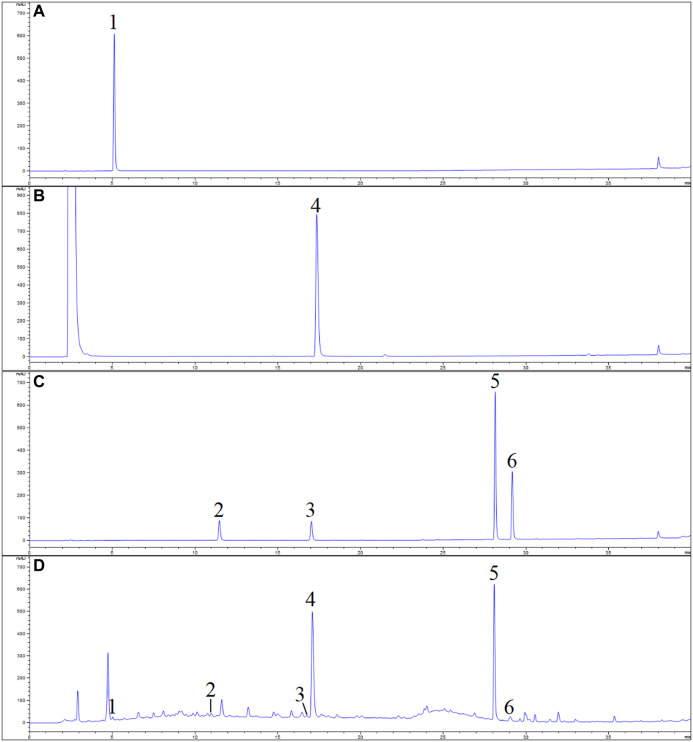
UPLC chromatogram of *Rubus chingii* var. *suavissimus* and the reference solutions **(A)** gallic acid; **(B)** ellagic acid; **(C)** mixed reference compounds; **(D)** sample. 1) Gallic acid, 2) caffeic acid, 3) rutin, 4) ellagic acid, 5) rubusoside, and 6) kaempferol.

**FIGURE 5:**
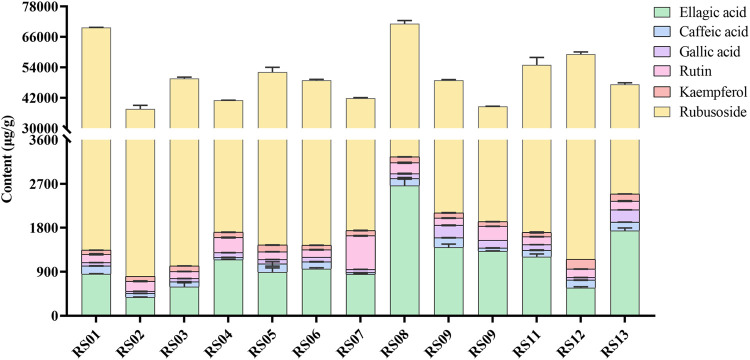
The content of ellagic acid, caffeic acid, gallic acid, rutin, kaempferol, and rubusoside in 13 batches of *Rubus chingii* var. *suavissimus* samples from markets.

The obtained peaks in the chromatograms were identified through the comparison of the retention times and on-line UV spectra with those of the standard constituents (Figure 4). Retention times of peaks 1–6 were 5.07, 11.03, 16.93, 17.29, 28.15, and 29.16 min, respectively. The results are summarized in Table 3 and Figure 5. The contents of gallic acid, caffeic acid, rutin, ellagic acid, rubusoside, and kaempferol in the samples ranged from 42.78 to 254.38 μg/g, 31.52 to 195.86 μg/g, 143.60 to 687.29 μg/g, 0.38 to 2.67 mg/g, 36.74 to 68.38 mg/g, and 93.28 to 140.45 μg/g, respectively.

**TABLE 3:** Content of six compounds in 13 batches of *Rubus chingii* var. *suavissimus* samples from markets (μg/g or mg/g, mean ± SD, *n = 3*).

No.	Collecting region	Sample type	Gallic acid (μg/g)	Caffeic acid (μg/g)	Rutin (μg/g)	Ellagic acid (mg/g)	Rubusoside (mg/g)	Kaempferol (μg/g)
RS01	Guilin, Guangxi	Tea bag	68.04±0.69	164.47±3.67	169.49±11.97	0.85±0.00	68.38±0.05	122.02±1.87
RS02	Guilin, Guangxi	Dried tea	42.78±1.29	77.08±2.02	206.33±8.60	0.38±0.00	36.85±1.37	95.45±2.97
RS03	Guilin, Guangxi	Tea bag	65.78±2.10	102.10±4.22	143.60±2.68	0.59±0.08	48.64±0.49	99.80±1.38
RS04	Guilin, Guangxi	Tea bag	97.11±2.80	44.98±3.55	308.58±7.51	1.15±0.01	39.34±0.02	93.28±0.92
RS05	Guilin, Guangxi	Dried tea	89.08±0.92	165.09±44.36	159.66±2.71	0.90±0.08	50.69±1.91	119.09±1.70
RS06	Laibin, Guangxi	Dried tea	91.52±1.05	141.40±4.40	153.33±5.51	0.96±0.02	47.46±0.40	94.83±2.30
RS07	Laibin, Guangxi	Dried tea	67.70±1.47	31.52±0.77	687.29±6.58	0.85±0.01	40.15±0.19	106.88±1.90
RS08	Laibin, Guangxi	Dried tea	101.25±8.10	141.71±11.47	223.89±10.08	2.67±0.16	67.92±1.23	120.68±2.41
RS09	Yongzhou, Hunan	Dried tea	254.38±6.22	195.86±4.71	148.78±5.19	1.40±0.07	46.77±0.33	106.41±3.68
RS10	Hezhou, Guangxi	Dried tea	155.21±0.37	64.74±3.70	287.72±2.35	1.32±0.10	36.74±0.02	110.66±4.42
RS11	Hezhou, Guangxi	Dried tea	113.47±1.40	132.34±7.10	161.06±4.18	1.21±0.06	53.28±2.91	140.45±3.84
RS12	Foshan, Guangdong	Dried tea	53.93±3.06	154.94±4.69	172.32±3.48	0.57±0.02	58.00±0.93	115.37±3.94
RS13	Yongzhou, Hunan	Dried tea	250.79±1.60	174.56±1.10	180.74±8.53	1.74±0.06	44.83±0.61	123.56±1.41

The results showed that the contents of the determined compounds varied in the RS samples obtained from different markets. For example, high levels of ellagic acid and rubusoside were present in all batches. However, even though the samples were purchased from the same origin, the contents could show significant differences. For example, while samples RS06∼ RS08 were all purchased from Laibin of Guangxi, the content of rubusoside and ellagic acid in these samples ranged from 40.15 to 67.92 mg/g and 0.85 to 2.67 mg/g, respectively. Similarly, the content of rubusoside and ellagic acid in samples RS01∼ RS05—obtained from Guilin—ranged from 36.85 to 68.38 mg/g and 0.38 to 1.15 mg/g, respectively. Importantly, the effectiveness of herbal medicine is highly related to the quality of the raw materials which on itself is affected by a complex set of factors such as the place of origin, growing conditions, medicinal parts, and processing methods. Previously, a higher content of rubusoside in tender tissues of RS (Yin et al., 2008) which was influenced by origin and dosage form (Fan et al., 2012) was shown.

In order to strengthen the quality control of RS, a strict control of the quality of raw materials from the origin and processing procedure is required. Thus, simultaneous determination methods to identify effective bioactive components could be of importance not only to analyze underlying mechanisms but also to establish future quality control of RS.

## Conclusion

In this study, a total of 69 compounds—including diterpenes, triterpenes, flavonoids, polyphenols, and lignans—were identified or tentatively characterized in *Rubus chingii* var. *suavissimus* by UPLC-Q/TOF-MS. Among them, 50 absorbed prototype components were detected in rat plasma. Next, bioactive compounds–therapeutic target network analysis predicted nine active components (ursolic acid, oleanolic acid, quercetin, kaempferol, gallic acid, ellagic acid, caffeic acid, rutin, and rubusoside), 71 target genes, and 20 pathways to be involved in RS-mediated alleviation of lipid metabolism disorder. Finally, UPLC analysis could show significant variability in the contents of six selected potential active compounds in RS samples obtained from different marketplaces. Taken together, the study provided a basis for further exploration of the mechanism on the regulation of lipid metabolism disorder and the development of *Rubus chingii* var. *suavissimus*–derived therapeutic drugs and healthcare products.

## Data Availability

The original contributions presented in the study are included in the article/Supplementary Material; further inquiries can be directed to the corresponding authors.
