# Supplementary material for: Integrating Constituents Absorbed into Blood, Network Pharmacology, and Quantitative Analysis to Reveal the Active Components in Rubus chingii var. suavissimus that Regulate Lipid Metabolism Disorder
Source: Front Pharmacol. 2021 Jun 29;12:630198. doi: 10.3389/fphar.2021.630198 (PMC8282055; doi:10.3389/fphar.2021.630198)
Supplement: Supplementary file 1 [file DataSheet1.docx]

# SUPPLEMENTARY MATERIALS

**Table S1.** Identification of absorbed compounds in plasma of rats after oral administration of *Rubus chingii* var. *suavissimus*.

| **No.** | **t_R_**  **(min)** | **Name** | **Formula** | **Theoretical mass (*m/z*)** | **Experimental mass (*m/z*)** | **Error**  **(ppm)** | **Fragmentations (*m/z*)** |
| --- | --- | --- | --- | --- | --- | --- | --- |
| P1* | 2.0 | Gallic acid | C_7_H_6_O_5_ | 169.0142 | 169.0143 | -0.5 | 125.0239 |
| P2 | 2.7 | Methyl gallate | C_8_H_8_O_5_ | 183.0299 | 183.0296 | 1.5 | 169.0121, 125.0238 |
| P3 | 2.7 | 2-pyrone-4,6-dicarboxylic acid | C_7_H_4_O_6_ | 182.9935 | 182.9922 | 7.3 | 139.0039,95.0160 |
| P7 | 6.5 | Caffeic acid-*O-*hexoside | C_15_H_18_O_9_ | 341.0878 | 341.0881 | -0.8 | 179.0339, 161.0426, 161.0265, 135.0435 |
| P8 | 7.2 | Quercetin-3,4’-*O*-*D*-*β*-glucopyranoside | C_27_H_30_O_17_ | 625.1410 | 625.1395 | 2.4 | 301.0333, 271.0265, 255.0313, 151.0039 |
| P9* | 7.2 | Caffeic acid | C_9_H_8_O_4_ | 179.0350 | 179.0348 | 0.7 | 135.0461 |
| P10 | 7.6 | Toringin | C_21_H_20_O_9_ | 415.1035 | 415.1049 | -3.5 | 161.0405, 151.0068 |
| P11 | 7.7 | Brevifolincarboxylic acid | C_13_H_8_O_8_ | 291.0146 | 291.0156 | -3.1 | 247.0278, 203.0320, 175.0376, 147.0430 |
| P13 | 8.7 | Ferulic acid-*O*-hexoside | C_16_H_20_O_9_ | 355.1035 | 355.1029 | 1.6 | 193.0501, 161.0425, 161.0265, 133.0263 |
| P16 | 11.2 | Cyanidin-3,5-diglucoside | C_27_H_30_O_16_ | 609.1461 | 609.1473 | -2.0 | 447.0973, 285.0430, 255.0312 |
| P17 | 11.2 | 5-hydroxy-7-methoxydihydroflavone | C_16_H_14_O_4_ | 269.0819 | 269.0821 | -0.5 | 145.0385, 105.0545 |
| P18* | 11.9 | Ellagic acid | C_14_H_6_O_8_ | 300.9990 | 300.9987 | 1.0 | 282.9892, 257.0038, 238.9957 |
| P19* | 12.2 | Rutin | C_27_H_30_O_16_ | 609.1461 | 609.1443 | 3.0 | 301.0354, 271.0286, 255.0310, 151.0041 |
| P20* | 12.4 | Hyperoside | C_21_H_20_O_12_ | 463.0882 | 463.0873 | 2.0 | 301.0346, 283.0237, 271.0262, 255.0311, 151.0048 |
| P21* | 12.7 | Isoquercitrin | C_21_H_20_O_12_ | 463.0882 | 463.0884 | -0.4 | 301.0360, 271.0262, 255.0272 |
| P22 | 13.5 | Cyanidin 3-*O*-rutinoside | C_27_H_30_O_15_ | 593.1512 | 593.1510 | 0.3 | 285.0390, 255.0305 |
| P23 | 14.2 | Guaijaverin | C_20_H_18_O_11_ | 433.0776 | 433.0776 | 0.1 | 301.0330, 283.0236, 255.0298, 151.0047 |
| P24 | 14.3 | Kaempferol-*O*-hexoside | C_21_H_20_O_11_ | 447.0933 | 447.0922 | 2.4 | 285.0403, 255.0305, 151.0042 |
| P25 | 14.6 | Kaempferol-3-*O*-rutinoside | C_27_H_30_O_15_ | 593.1512 | 593.1508 | 0.7 | 285.0391, 255.0310, 151.0057 |
| P26* | 15.3 | Quercitrin | C_21_H_20_O_11_ | 447.0933 | 447.0927 | 1.4 | 283.0233, 271.0266, 255.0209, 151.0028 |
| P28 | 17.8 | Luteoloside | C_21_H_20_O_11_ | 447.0933 | 447.0935 | -0.5 | 285.0428, 255.0309, 151.0018 |
| P32* | 22.3 | Quercetin | C_15_H_10_O_7_ | 301.0354 | 301.0346 | 2.6 | 283.0230, 271.0235, 255.0306, 151.0023 |
| P33 | 23.6 | glaucocalyxin G | C_26_H_42_O_10_ | 513.2705 | 513.2703 | 0.4 | 351.2181, 333.2054 |
| P36 | 24.6 | 7*β*-hydroxysteviol | C_20_H_30_O_4_ | 333.2071 | 333.2065 | 2.0 | 315.2158, 289.2126, 271.2021 |
| P37 | 24.7 | Cussovantoside A | C_26_H_42_O_10_ | 513.2705 | 513.2702 | 0.7 | 351.2160, 333.2102, 271.2048 |
| P39 | 25.6 | Sugereoside | C_26_H_42_O_8_ | 481.2807 | 481.2805 | 0.3 | 319.2241, 301.2251 |
| P40 | 26.0 | 7*β*,17-dihydroxy-ent-kaur-15-en-19-oic acid 19-*O*-*β*-*D*-glucopyranoside ester | C_26_H_40_O_9_ | 495.2600 | 495.2593 | 1.4 | 333.2067, 285.1899 |
| P41 | 26.0 | 7*β*,17-dihydroxy-16*β*-ent-kauran-19-oic acid 19-*O*-*β*-*D*-glucopyranoside ester | C_26_H_42_O_9_ | 497.2756 | 497.2748 | 1.7 | 335.2209, 317.2105 |
| P43 | 26.4 | ent-3*α*,16*β*,17-trihydroxy-kauran-19-oic acid | C_20_H_32_O_5_ | 351.2177 | 351.2171 | 1.6 | 333.2060, 303.1989 |
| P44* | 26.6 | Kaempferol | C_15_H_10_O_6_ | 285.0405 | 285.0402 | 0.9 | 255.0304, 151.0060 |
| P45 | 26.6 | Suavisoside-A | C_26_H_44_O_8_ | 483.2963 | 483.2963 | 0.1 | 321.2397, 285.2231 |
| P47 | 28.0 | ent-13,17-dihdyroxy-kauran-15-en-19-oic acid | C_20_H_30_O_4_ | 333.2071 | 333.2071 | 0.0 | 315.1983, 289.2181, 271.2047 |
| P48 | 28.0 | 13-[(*O*-*β*-*D*-glucopyranosyl)oxy] ent-kaur-16-en-19-oic acid-2-*O*-*β*-*D*-glucopyranosyl-*β*-*D*-  glucopyranosyl ester | C_38_H_60_O_18_ | 803.3707 | 803.3611 | -0.4 | 641.3174, 479.2671, 317.2106 |
| P49 | 28.2 | 17-*O*-*β*-*D*-glucopyranosyl-16*α*-ent-kauran-19-oic acid | C_26_H_42_O_8_ | 481.2807 | 481.2802 | 1.0 | 319.2242, 301.2264, |
| P50* | 28.4 | Rubusoside | C_32_H_50_O_13_ | 641.3179 | 641.3186 | -1.1 | 479.2666, 317.2124, 273.2219 |
| P51 | 29.1 | 16*α*,17-dihydroxy-kauran-19-oic acid | C_20_H_32_O_4_ | 335.2228 | 335.2225 | 0.9 | 335.2246, 291.2295 |
| P52 | 29.1 | Ganoderic acid C2 | C_30_H_46_O_7_ | 517.3171 | 517.3168 | 0.5 | 499.3049, 481.2909, 473.3269, 469.3001 |
| P53 | 29.1 | Platycodigenin | C_30_H_48_O_7_ | 519.3327 | 519.3322 | 1.0 | 501.3127, 471.3114, 459.3132 |
| P54 | 29.4 | Terminolic acid | C_30_H_48_O_6_ | 503.3378 | 503.3379 | -0.1 | 485.3256, 473.3245 |
| P55 | 30.1 | 2*α*,3*β*,19*α*,23-tetrahydroxy-urs-12-en-28-oic acid | C_30_H_48_O_6_ | 503.3378 | 503.3377 | 0.3 | 485.3282, 473.3354, 455.3105 |
| P56 | 30.1 | ent-16*α*,17-dihydroxy-kauran-19-oic acid | C_20_H_32_O_4_ | 335.2228 | 335.2222 | 1.7 | 317.2131, 291.2307 |
| P58 | 30.4 | Paniculoside Ⅳ | C_26_H_42_O_9_ | 497.2756 | 497.2762 | -1.2 | 335.2241, 317.2115 |
| P59 | 30.4 | ent-kauran-16-en-19-oic-13-*O*-*β*-*D*-glucoside | C_26_H_40_O_8_ | 479.2650 | 479.2651 | -0.1 | 317.2128, 273.2121, 255.2310 |
| P61 | 30.8 | Ilexgenin A | C_30_H_46_O_6_ | 501.3222 | 501.3221 | 0.1 | 483.3130, 467.3088 |
| P62 | 31.0 | Cussoracosides E | C_26_H_40_O_8_ | 479.2650 | 479.2664 | -2.9 | 317.2110, 273.2243 |
| P64* | 34.6 | Steviol | C_20_H_30_O_3_ | 317.2122 | 317.2119 | 1.0 | 273.2202, 255.2186 |
| P65 | 34.9 | ent-16*β*, 17-dihydroxy-kauran-3-one | C_20_H_32_O_3_ | 319.2279 | 319.2272 | 2.0 | 289.2202, 273.2267 |
| P66* | 35.3 | Isosteviol | C_20_H_30_O_3_ | 317.2122 | 317.2119 | 0.9 | 273.2218, 255.2149 |
| P68* | 38.7 | Ursolic acid | C_30_H_48_O_3_ | 455.3531 | 455.3521 | 2.2 | 411.3442, 393.3532 |
| P69* | 39.0 | Oleanolic acid | C_30_H_48_O_3_ | 455.3531 | 455.3522 | 1.9 | 411.3429, 393.3531 |

Note: * the compound was identified by comparing with reference substances, P: prototype.

# Table S2. Details of the top 20 results of KEGG pathway enrichment from DAVID

| No. | Term | *p*-value | Count | Gene |
| --- | --- | --- | --- | --- |
| 1 | hsa04931: Insulin resistance | 7.02E-^08^ | 11 | SREBP1, PIK3CA, NOS3, NR1H2, INSR, NR1H3, AKT1, PTPN11, PPARα, TNF, MTOR |
| 2 | hsa03320: PPAR signaling pathway | 2.18E^-07^ | 9 | FABP2, AP2, SCD, APOA2, LPL, NR1H3, PPARγ, PPARα, PPARD |
| 3 | hsa04152: AMPK signaling pathway | 2.49E^-06^ | 10 | SREBP1, PIK3CA, SCD, INSR, AKT1, PPARγ, HMGCR, CFTR, MTOR, ACC1 |
| 4 | hsa04066: HIF-1 signaling pathway | 3.55E^-06^ | 9 | PIK3CA, NOS3, INSR, SERPINE1, MAPK1, AKT1, TLR4, MTOR, VEGFA |
| 5 | hsa00140: Steroid hormone biosynthesis | 1.85E^-05^ | 7 | HSD11B1, CYP1A2, CYP1B1, COMT, CYP3A4, CYP19A1, CYP17A1 |
| 6 | hsa05205: Proteoglycans in cancer | 2.02E^-05^ | 11 | PIK3CA, FAS, MAPK1, AKT1, PTPN11, ESR1, TNF, TP53, TLR4, MTOR, VEGFA |
| 7 | hsa00980: Metabolism of xenobiotics by cytochrome P450 | 7.50E^-05^ | 7 | HSD11B1, CYP2C9, CYP2D6, GSTP1, CYP1A2, CYP1B1, CYP3A4 |
| 8 | hsa04913: Ovarian steroidogenesis | 1.03E^-04^ | 6 | ALOX5, INSR, CYP1B1, PTGS2, CYP19A1, CYP17A1 |
| 9 | hsa05204: Chemical carcinogenesis | 1.16E^-04^ | 7 | HSD11B1, CYP2C9, GSTP1, CYP1A2, CYP1B1, CYP3A4, PTGS2 |
| 10 | hsa04370: VEGF signaling pathway | 2.94E^-04^ | 6 | PIK3CA, NOS3, MAPK1, AKT1, PTGS2, VEGFA |
| 11 | hsa05230: Central carbon metabolism in cancer | 3.69E^-04^ | 6 | G6PD, PIK3CA, MAPK1, AKT1, TP53, MTOR |
| 12 | hsa00982: Drug metabolism - cytochrome P450 | 4.89E^-04^ | 6 | CYP2C9, MAOA, CYP2D6, GSTP1, CYP1A2, CYP3A4 |
| 13 | hsa04920: Adipocytokine signaling pathway | 5.59E^-04^ | 6 | AKT1, PTPN11, JAK2, PPARα, TNF, MTOR |
| 14 | hsa04668: TNF signaling pathway | 5.69E^-04^ | 7 | PIK3CA, FAS, MAPK1, AKT1, PTGS2, SELE, TNF |
| 15 | hsa04917: Prolactin signaling pathway | 5.97E^-04^ | 6 | PIK3CA, MAPK1, AKT1, JAK2, ESR1, CYP17A1 |
| 16 | hsa04932: Non-alcoholic fatty liver disease (NAFLD) | 6.17E^-04^ | 8 | SREBP1, PIK3CA, INSR, FAS, NR1H3, AKT1, PPARα, TNF |
| 17 | hsa04726: Serotonergic synapse | 6.91E^-04^ | 7 | CYP2C9, APP, MAOA, CYP2D6, ALOX5, MAPK1, PTGS2 |
| 18 | hsa04930: Type II diabetes mellitus | 0.001159 | 5 | PIK3CA, INSR, MAPK1, TNF, MTOR |
| 19 | hsa05200: Pathways in cancer | 0.001302 | 12 | AR, PIK3CA, GSTP1, FAS, MAPK1, AKT1, PPARγ, PTGS2, TP53, MTOR, PPARD, VEGFA |
| 20 | hsa04923: Regulation of lipolysis in adipocytes | 0.002064 | 5 | aP2, PIK3CA, INSR, AKT1, PTGS2 |

**Table S3.** The regression equation, Linearity range, LOQs and LODs of six analytes. (*n = 6*)

| **Analyte** | **Regression equation** | ***r*^2^** | **Linearity range(μg/mL)** | **LOD (ng)** | **LOQ (ng)** |
| --- | --- | --- | --- | --- | --- |
| Gallic acid | *Y*=0.00008*X* – 0.0056 | 0.9993 | 2.140~1072 | 0.40 | 1.00 |
| Caffeic acid | *Y*=0.0002*X* + 0.0004 | 0.9999 | 0.3078~153.9 | 0.30 | 1.00 |
| Rutin | *Y*=0.0002*X* + 0.0002 | 1.0000 | 0.3096~154.8 | 0.41 | 1.25 |
| Ellagic acid | *Y*=0.00005*X* – 0.0016 | 0.9999 | 1.139~1139 | 0.40 | 1.20 |
| Rubusoside | *Y*=0.0007*X* – 0.0109 | 0.9999 | 9.910~4955 | 1.00 | 3.00 |
| Kaempferol | *Y*=0.00005*X* + 0.0002 | 0.9999 | 0.2320~92.80 | 0.09 | 0.30 |

**Table S4.** Precision, repeatability, stability, recovery of six analytes. (*n = 6*)

| **Analytes** | **Precision** | | **Repeatability**  **RSD (%)** | **Stability**  **RSD (%)** | **Recovery** | | | | |
| --- | --- | --- | --- | --- | --- | --- | --- | --- | --- |
|  | **Intra-day**  **RSD (%)** | **Inter-day RSD (%)** |  |  | **Contained (mg)** | **Added**  **(mg)** | **Detected (mg)** | **Recovery (%)** | **RSD**  **(%)** |
| Gallic acid | 1.50 | 1.38 | 1.64 | 1.89 | 0.02 | 0.02 | 0.04 | 97.58 | 1.29 |
| Caffeic acid | 1.45 | 1.93 | 1.75 | 0.74 | 0.09 | 0.09 | 0.19 | 98.59 | 1.30 |
| Rutin | 0.64 | 1.53 | 2.94 | 1.59 | 0.08 | 0.08 | 0.16 | 98.79 | 1.30 |
| Ellagic acid | 0.37 | 0.38 | 2.46 | 0.74 | 1.38 | 1.37 | 2.81 | 99.70 | 2.24 |
| Rubusoside | 0.93 | 0.69 | 1.89 | 0.16 | 12.37 | 12.33 | 24.98 | 99.93 | 2.31 |
| Kaempferol | 0.53 | 1.87 | 2.85 | 0.94 | 0.05 | 0.05 | 0.10 | 100.29 | 1.21 |

# FIGURE LEGENDS

**

**

**Figure S1.** The structure of the 69 identified and tentatively characterized compounds in *Rubus chingii* var. *suavissimus*.

**

**

**Figure S2.** The detailed fragmentation pathway of (A) rubusoside, (B) rutin, and (C) corilagin.
